# Urgent thyroidectomy in the immediate post-lung transplant period

**DOI:** 10.1093/jscr/rjaf655

**Published:** 2025-08-26

**Authors:** Jaime L Sepulveda, Alessia C Cioci, John I Lew

**Affiliations:** DeWitt Daughtry Family Department of Surgery, University of Miami Leonard M. Miller School of Medicine, 1600 NW 10th Avenue, Miami, FL 33136, United States; DeWitt Daughtry Family Department of Surgery, University of Miami Leonard M. Miller School of Medicine, 1600 NW 10th Avenue, Miami, FL 33136, United States; DeWitt Daughtry Family Department of Surgery, University of Miami Leonard M. Miller School of Medicine, 1600 NW 10th Avenue, Miami, FL 33136, United States

**Keywords:** lung transplant, thyroid cancer, thyroid nodule, transplant surgery, total thyroidectomy

## Abstract

Transplant eligibility in patients with suspected or untreated malignancy presents a complex clinical dilemma. Transplant guidelines historically recommend a cancer-free interval of 2-to-5 years prior to solid organ transplantation to reduce the risk of recurrence under immunosuppression. However, these timelines are not always feasible. We present the case of a woman with end-stage lung disease found to have a thyroid nodule with intermediate-high risk of malignancy. Due to her severe respiratory illness, she was deemed unlikely to meet the recommended cancer-free interval. After multidisciplinary evaluation, the patient underwent bilateral lung transplantation prior to thyroidectomy. Concern for tumor progression under immunosuppression led to total thyroidectomy in the immediate postoperative period. This case highlights the need for flexible transplant evaluation pathways and presents a rare instance of non-emergent oncologic surgery performed in the immediate post-transplant period. This work contributes to a growing body of literature advocating for nuance in transplant oncology decision-making.

## Introduction

The presence of active or recently treated malignancy in candidates for solid organ transplantation presents a significant clinical challenge due to the heightened risk of tumour recurrence under post-transplant immunosuppression. Historically, transplant eligibility has required a cancer-free interval of 2 to 5 years, depending on tumour type and stage [1]. However, recent consensus statements from the International Society for Heart and Lung Transplantation and the American Society of Transplantation advocate for a more individualized approach accounting for tumour biology, recurrence rates, and overall oncologic prognosis [2].

Differentiated thyroid carcinoma, notable for its more indolent behaviour and excellent long-term outcomes, is increasingly considered a potential exception to fixed disease-free intervals [3]. However, no disease-specific guidelines exist regarding the timing of transplantation in patients with suspected or untreated thyroid malignancy, and many transplant centers remain cautious in such scenarios.

We report a case involving a patient with end-stage lung disease and a thyroid nodule suspicious for malignancy who underwent bilateral lung transplantation followed by total thyroidectomy in the immediate postoperative period.

## Case presentation

A 59-year-old woman with end-stage lung disease secondary to interstitial lung disease in the setting of juvenile rheumatoid arthritis was evaluated for bilateral lung transplantation. Pre-transplant imaging revealed a 0.8 cm indeterminate left thyroid nodule. Fine-needle aspiration cytology was classified as Bethesda category III (atypia of undetermined significance), and molecular testing via ThyroSeq identified an NRAS mutation associated with a 70% risk of malignancy.

Due to her severe pulmonary disease requiring 4 L of supplemental oxygen at rest, she was deemed a high-risk surgical candidate for thyroidectomy. Pulmonary function testing demonstrated severe restrictive lung disease. Given the urgent need for transplantation and the indolent behaviour of differentiated thyroid neoplasms, the transplant and endocrine surgery teams elected to proceed with bilateral lung transplantation prior to definitive thyroid surgery.

Following an uncomplicated bilateral lung transplant, the patient was initiated on standard triple immunosuppressive therapy. Due to concern for immunosuppression-induced tumor progression, the transplant team recommended early resection of the thyroid nodule. After multidisciplinary discussion, a total thyroidectomy was performed on post-transplant Day 5.

The thyroidectomy was uncomplicated. Final pathology demonstrated a benign follicular adenoma with focal oncocytic (Hurthle cell) changes, measuring 0.4 cm at its greatest dimension, with no evidence of malignancy. The patient’s postoperative course was complicated by hypoxic respiratory failure requiring tracheostomy and pericardial effusion requiring pericardiocentesis, but she improved steadily and was transferred to inpatient rehabilitation in stable condition on post-transplant Day 33.

## Discussion

Transplant eligibility in patients with suspected or active malignancy remains a challenging area of clinical decision-making. Traditional guidelines have emphasized the need for a cancer-free interval of at least 2–5 years prior to solid organ transplantation, largely to mitigate the risk of accelerated tumor progression under immunosuppression. These recommendations are increasingly questioned considering emerging evidence and consensus guidelines supporting individualized assessment based on tumor biology, recurrence risk, and clinical urgency [1, 2].

Thyroid nodules with indeterminate cytology, particularly Bethesda III lesions, present a unique challenge in transplant candidates. In this case, the presence of an NRAS proto-oncogene mutation and a reported 70% risk of malignancy would warrant timely surgical resection. However, the patient’s severe pulmonary disease rendered her a poor surgical candidate at the time of evaluation, and delay in lung transplantation for the recommended disease-free interval would likely lead to death from respiratory failure. This case therefore exemplifies a tension in transplant oncology: balancing the morbidity of immediate intervention against the oncologic risk of deferral under immunosuppression.

The literature regarding the management of thyroid cancer in transplant candidates is limited. While differentiated thyroid cancer is generally associated with favorable long-term survival and low recurrence rates, most transplant centers require either evidence of remission for at least 2–5 years or a completed oncologic workup prior to listing [4]. There is little precedent for proceeding with transplantation in the setting of an untreated, suspicious thyroid lesion. A 2024 review in the New England Journal of Medicine emphasizes a case-by-case approach in such scenarios, but does not offer specific guidance for the management of indeterminate thyroid nodules pre-transplant [1]. A review by Webb *et al.* (2021) notes that survival outcomes and recurrence rates in patients with thyroid cancer are not significantly affected by solid organ transplantation, indicating that the discovery of suspicious thyroid nodules during transplant evaluation should not preclude transplant listing [3]. The literature is even more limited in describing early postoperative oncologic surgery, particularly within the first week after solid organ transplant. Surgery in the immediate postoperative period following bilateral lung transplantation is generally avoided unless there is a compelling, urgent indication, with the main risks being the profound immunosuppression required to prevent graft rejection increasing the risk of surgical site and systemic infections, impaired wound healing, and elevated risk of perioperative complications such as dehiscence and poor tissue repair [5]. The decision to proceed with thyroidectomy on post-operative Day 5 was driven by concern regarding the potential for immunosuppression-induced tumour growth, an area where clinical evidence remains sparse. The impact of immunosuppression on thyroid cancer remains unclear, though studies suggest a complex relationship [6].

This case highlights several key messages. First, it underscores the need for multidisciplinary, nuanced decision-making when transplant eligibility intersects with oncologic uncertainty. Second, it demonstrates that with careful coordination, non-transplant surgery can be safely performed in the immediate post-transplant period in select cases. Finally, it raises the question of whether existing transplant guidelines sufficiently accommodate the spectrum of oncologic risk presented by different malignancies, particularly those such as differentiated thyroid cancer that often behave indolently. While the final pathology in this case revealed a benign follicular adenoma, the decision-making framework and the timing of surgery remain clinically relevant given the high preoperative suspicion for malignancy.

This case supports the growing movement toward individualized transplant oncology assessment, while also contributing to a limited body of literature on early post-transplant non-emergent surgery. It reinforces the importance of weighing immediate survival benefits of transplantation against potential oncologic risks and may serve as a reference point for similar decisions in future multidisciplinary transplant evaluations.

## References

[1] Christie JD, Van Raemdonck D, Fisher AJ. Lung transplantation. N Engl J Med 2024;391:1822–36. 10.1056/NEJMra2401039 39536228

[2] Catelli C, Faccioli E, Silvestrin S, et al. Lung transplantation in patients with previous or unknown oncological disease: evaluation of short- and long-term outcomes. Cancers (Basel) 2024;16:538. 10.3390/cancers16030538 38339288 PMC10854809

[3] Webb C, Cronin P, Gupta N, et al. Is thyroid cancer prognosis affected by solid organ transplantation? Surgery (United States) 2021;169:58–62. 10.1016/j.surg.2020.06.044 32814633

[4] Stenman C, Wallinder A, Holmberg E, et al. Malignancies after lung transplantation. Transpl Int 2024;37:12127. 10.3389/ti.2024.12127 PMC11417467 39314925

[5] Padiyar J . Critical care considerations in the post-operative period for the lung transplant patient. J Thorac Dis 2021;13:6747–53. 10.21037/jtd-21-1441 34992850 PMC8662514

[6] Grulich AE, van Leeuwen MT, Falster MO, et al. Incidence of cancers in people with HIV/AIDS compared with immunosuppressed transplant recipients: a meta-analysis. Lancet 2007;370:59–67. 10.1016/S0140-6736(07)61050-2 17617273

